# Dominance of dengue virus serotype-2 in Pakistan (2023–2024): Molecular characterization of the envelope gene and exploration of antiviral targets

**DOI:** 10.1016/j.virusres.2024.199497

**Published:** 2024-11-23

**Authors:** Haidar Ali, Iffat Saleem, Muhammad Saad Ahmed, Deeba Amraiz, Imran Shahid, Eman A. Al-Shahari, Jing Yang, Liaqat Ali

**Affiliations:** a Department of Biological Sciences, National University of Medical Sciences (NUMS), Rawalpindi, Pakistan; b Department of Pharmacology & Toxicology, Faculty of Medicine, Umm Al-Qura University, Al-Abidiyah, P.O. Box 13578, Makkah, 21955, Saudi Arabia; c Department of Biology, Applied College Muhayil Assir, King Khalid University, Abha, Saudi Arabia; d Wuhan Institute of Biological Products Co., Ltd., Wuhan, Hubei, China

**Keywords:** Dengue virus, *Flaviviridae*, *Aedes aegypti*, Envelope gene, Dengue shock syndrome, Dengue hemorrhagic fever, Arbidol, Quercetin, BepiPred-3.0

## Abstract

Dengue virus infection, caused by a single positive-stranded RNA virus from the *Flaviviridae* family, represents a significant public health challenge in tropical and subtropical regions. This virus has four serotypes (DENV-1, 2, 3, and 4), primarily transmitted by Aedes mosquitoes. Despite extensive research, effective antiviral treatments and vaccines remain elusive due to the viral diversity and the complex mechanisms such as antibody-dependent enhancement (ADE). In the current study, NS1-positive serum samples from dengue cases in Pakistan (2023–2024), were analyzed to determine the predominant serotype and characterize the envelope (E) gene for further exploration of antiviral targets. Out of 100 samples, 63 (63%) tested positive for DENV-2, indicating its predominance during this period, while two samples showed mixed infections with DENV-2 and DENV-3. The envelope gene was successfully amplified using nested PCR, validated through gel electrophoresis and sanger sequencing. Phylogenetic analysis revealed high similarity of the DENV-2 isolates to strains from China and India. Computational modeling of the envelope protein structure identified potential antiviral binding sites and further molecular docking studies suggested that specific antiviral compounds like Arbidol and Quercetin can inhibit early steps in viral infection. Additionally, BepiPred-3.0 predicted several B-cell epitopes, which could be useful for vaccine development. These findings enhance our understanding of dengue epidemiology in Pakistan and contribute to the development of targeted antiviral therapies, potentially informing future vaccination strategies and outbreak management.

## Highlights

• Identified the predominant dengue virus serotype (DENV-2) circulating in Pakistan during 2023–2024.
• Characterization of the envelope protein that provided valuable insights into the virus molecular structure and function.
• The high sequence similarity to DENV-2 strains from different regions underscores the widespread distribution and genetic stability of this serotype.
• Computational docking studies with antiviral compounds like Arbidol and Quercetin indicated potential therapeutic interactions.

## 1 Introduction

Dengue virus causes dengue fever, poses a serious health threat, especially in tropical regions (Ali et al., 2022). It belongs to the family *Flaviviridae* and has four distinct serotypes. The *Aedes aegypti* mosquito, which is active during the day, is the primary vector of transmission, with the *Aedes albopictus* mosquito serving as a secondary transmitter. (Ali et al., 2022; Guo et al., 2017; Harapan et al., 2020; Yousaf et al., 2018). The infection can cause a variety of clinical manifestations, ranging from mild undifferentiated fever to more severe forms, such as Dengue Hemorrhagic Fever (DHF) and Dengue Shock Syndrome (DSS) (Ng et al., 2022).

It has a substantial global burden and spreads widely. Dengue affects 400 million people annually, of whom 96 million live in developing nations. The disease causes noticeable symptoms and about 21,000 deaths, most often in tropical and subtropical regions. (Bhatt et al., 2013; Murugesan and Manoharan, 2020). Dengue fever is a serious public health issue in Pakistan, where there have been multiple outbreaks in different cities throughout the nation. The country has seen a notable increase in dengue cases, with the worst epidemic occurring in the Punjab province in 2011 and resulting in over 286,857 suspected cases, 21,685 confirmed cases, and 350 deaths. (Usman, 2018). The dynamic nature of DENV circulation in the area has been highlighted by seroprevalence studies and outbreak investigations, with changes in the most common serotypes noted over time. In 2017, DENV-3 replaced DENV-1 as the most common serotype in India, indicating continued viral evolution and changes in the epidemiology. (Patil et al., 2018). Comparably, during outbreaks, Pakistan has seen the co-circulation of several DENV serotypes, with variations in the predominant strains recorded over time.. Studies carried out in different parts of Pakistan have identified serotypes like DENV-1, DENV-2, and DENV-3 as the main agents responsible, highlighting the necessity of effective surveillance and control protocols. (Fatima et al., 2011; Khan et al., 2008).

The single-stranded RNA genome that makes up dengue virus, also referred to as DENV, is approximately 10.6 kilobases (kb) long (Ali et al., 2022). Three structural proteins, such as the capsid, envelope, and membrane, and seven non-structural proteins, such as NS1, NS2A, NS2B, NS3, NS4A, NS4B, and NS5, make up the single-stranded positive RNA genome of DENV (Dethoff et al., 2018). The capsid (C), membrane (prM/M), and envelope (E) proteins of DENV constitute the virus's structural components and are essential for the virus's entrance, assembly, and fusion with host cells. These proteins exhibit complex interactions with viral and host factors orchestrating key steps in the virus lifecycle (Mukhopadhyay et al., 2005; Zhang et al., 2013). The non-structural proteins of the dengue virus are also pivotal to its life cycle, contributing to processes such as RNA replication, virion assembly, immune modulation, and pathogenesis. Among these, NS1 is essential for virion production, while NS5 plays a key role in RNA replication and disrupting the host immune response, Gaining a deeper understanding of these functions offers valuable insights for developing therapeutic targets and vaccine strategies (Bhatnagar et al., 2021; Scaturro et al., 2015).

The envelope gene of the dengue virus encodes the E protein, a key element of the virus outer envelope, it is essential for various critical functions including the formation of new virions, attachment to cell receptors and fusion of membranes (Chambers et al., 1990; Modis et al., 2005; Roehrig et al., 1989). There are three domains in the E protein: E-DI, E-DII, and E-DIII (Zhang et al., 2012, 2004). Crystal structure studies have elucidated the arrangement of these domains in both the dimeric pre-fusion conformation and the low-pH-induced post-fusion trimer (Bressanelli et al., 2004; Klein et al., 2013). E-DI, the N-terminal domain, is structurally central, followed by E-DII, the dimerization domain, and E-DIII, the C-terminal immunoglobulin-like domain. Adjacent to E-DIII is the α-helical stem-anchor region (Zhang et al., 2003). Additionally, endoplasmic reticulum-resident chaperones like calnexin, calreticulin, and BiP interact with envelope protein. These chaperones are essential for the correct folding and assembly of viral proteins, which promotes virus production. (Limjindaporns et al., 2009). Targeting conserved pockets on these proteins could lead to broad-spectrum antivirals against various mosquito-borne *flaviviruses* (de Wispelaere et al., 2018).

The primary goals of the current study were to amplify and characterize the dengue virus's envelope gene in order to learn more about its evolutionary dynamics, genetic diversity, and functional importance. In order to address this urgent global health issue, it was aimed to contribute to the development of innovative methods for diagnosing, preventing, and treating dengue fever by clarifying the molecular characteristics of the envelope gene.

## 2 Methods

### 2.1 Sample collection

Samples were collected using a random sampling technique. A total of 100 DENV NS1-positive serum samples were obtained from various hospital laboratories in the twin cities (Islamabad and Rawalpindi) of Pakistan from February 2023 to March 2024 and were stored at -80 °C for further processing. These samples represented both mild and severe dengue cases and were collected during the acute phase of infection (days 3–7 post-onset of symptoms).

### 2.2 RNA extraction, cDNA synthesis & serotyping

RNA was extracted from DENV samples using the NucleoSpin RNA Extraction Kit (Macherey-Nagel, Germany) according to the manufacturer's protocol. Using a Nanodrop spectrophotometer (Thermo Fisher Scientific), absorbance measurements at 260 and 280 nm were used to determine the quality and quantity of extracted RNA. RevertAid First Strand cDNA Synthesis Kit from (Thermo Scientific™) was used to synthesize the cDNA from RNA of DENV. For serotyping, a protocol developed by our collaborators at Molecular Virology and Diagnostics Lab at the Centre of Excellence in Molecular Biology (CEMB), University of Punjab, Lahore, was utilized. The process involved two rounds of conventional PCR using Serotype-specific primers. To identify the dengue virus genome, universal primers (D1-D and D2-D) that target the C-PrM junction were utilized (Fatima et al., 2011, Lanciotti et al., 1992). To identify DENV serotypes, four sets of type-specific primers (TS1-F/R, TS2-F/R, TS3-F/R, and TS4-F/R) were employed (Fatima et al., 2011). The primers used for this purpose are listed in Table 1. The amplified products were then visualized using gel electrophoresis on a 1% agarose gel, and the bands were observed under UV light.

**Table 1:** The List of Primers for Amplification of C-PrM region for DENV Serotyping (Fatima et al., 2011).

S. No.	5′-3′ Sequence	Primer Name	Fragments Size in Base pairs
1	TCAATATGCTGAAACGCGWGAGAAACCG	D1-D	511 bp
2	TTGCACCARCARTCWATGTCTTCWGGYTC	D2-D	
3	AGGACCCATGAAATTGGTGA	TS1-Forward	411 bp
4	ACGTCATCTGGTTCCGTCTC	TS1-Reverse	
5	AGAGAAACCGCGTGTCAACT	TS2-Forward	403 bp
6	ATGGCCATGAGGGTACACAT	TS2- Reverse	
7	ACCGTGTGTCAACTGGATCA	TS3- Forward	453 bp
8	CAGTAATGAGGGGGCATTTG	TS3- Reverse	
9	CCTCAAGGGTTGGTGAAGAG	TS4- Forward	401 bp
10	CCTCACACATTTCACCCAAGT	TS4- Reverse	

### 2.3 Amplification of DENV structural gene (envelope)

Two rounds of nested PCR to amplify the structural Envelope gene was used to obtain the gene fragments. All primers for this process were optimized. Upon completing the optimization, the protocol for amplifying the Envelope gene was finalized. The primers utilized for this purpose are specified in Table 2.

**Table 2:** The List of Primer for the amplification of Envelope gene of Dengue Virus.

Structural Gene (Envelope) —–1.4 kb (1485 bps)			
S. No.	Primers	5′-3′ Sequence	Round
1	Envelope-OF	TGGCATACACCATAGGAACG	Round 1st
2	Envelope -IF	TGCGAAGAAACAGGATGTTG	Round 2nd
3	Envelope -OR	GGGGATTCTGGTTGGAATTT	Round 1st
4	Envelope -IR	AAATCCCACTGCCACATTTC	Round 2nd

### 2.4 DNA purification for sequencing

The PCR product was purified from the second round using a DNA purification kit (SolarBio), that utilizes a silica membrane to isolate DNA fragments between 100 bp and 40 kb with an 80% recovery rate. The process involved mixing 30 µL of the PCR product with 150 µL of binding buffer and transferring the mixture to an adsorption column. After incubation and centrifugation, the DNA was washed twice with ethanol-based washing buffer and air-dried. Finally, the DNA was eluted with 35 µL of pre-heated elution buffer and stored at -20 °C for further use.

### 2.5 Sequencing

Purity of the DNA products was confirmed through Sanger sequencing. Gene-specific primers, including both forward and reverse, were utilized to sequence both strands of the transcripts. Sequencing was conducted by Macrogen in South Korea, with services facilitated by Alpha Genomics Lab. The resulting sequences were then analyzed using the NCBI BLAST tool (https://blast.ncbi.nlm.nih.gov/Blast.cgi) to for verification before being submitted to GenBank, where they were assigned the accession number PP594161.

### 2.6 *Insilico* characterization

#### 2.6.1 Multiple Sequence Alignment (MSA)

The multiple sequence alignment (MSA) of nucleotide sequences was conducted using the T-Coffee Server (https://tcoffee.crg.eu/), incorporating DENV-2 sequences isolated in our study (PP594161) and various GenBank entries from different serotypes (GenBank no. MZ26820, MZ27530, MZ31055, MZ31056) to identify evolutionary relationships and mutational patterns.

#### 2.6.2 Phylogenetic analysis

Phylogenetic analysis of the sequences was conducted using MEGA 11, employing the Neighbor-Joining method. A phylogenetic tree was constructed based on the distance matrix, with 100 bootstrap replications to assess the robustness of the tree. The analysis incorporated sequences isolated in our study from different regions of Pakistan, along with additional sequences obtained through BLAST searches, providing insights into the genetic relationships among the DENV-2 strains.

#### 2.6.3 Homology modeling

The amino acid sequences were predicted from nucleotide sequences using ExPASy Translate tool (https://web.expasy.org/translate/), and the 3D structure of the envelope protein was modeled using Swiss-Model Server (https://swissmodel.expasy.org/) then PyMOL was utilized for visualization.

#### 2.6.4 Molecular docking

The 3D structures of Arbidol and Quercetin were retrieved from PubChem (https://pubchem.ncbi.nlm.nih.gov/). Molecular docking with the dengue serotype 2 envelope protein sequence obtained from our study was performed using CB-Dock (http://clab.labshare.cn/cb-dock/), and the binding affinities and interactions were visualized.

#### 2.6.5 Epitope prediction

BepiPred-3.0 (https://services.healthtech.dtu.dk/services/BepiPred-3.0/) was used for B-cell epitope prediction, utilizing sequence-based analysis for all DENV serotypes.

## 3 Results

### 3.1 Serotyping of Dengue Positive Samples

**Fig. 1:**
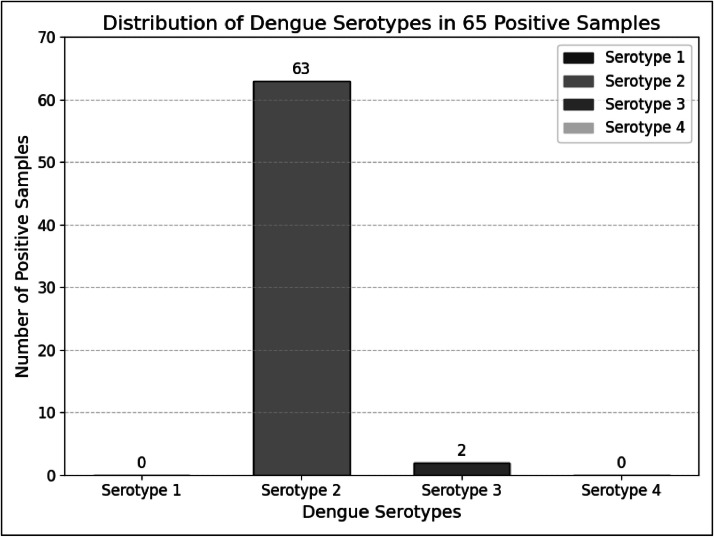
Bar graph showing the distribution of dengue serotypes in 65 positive samples.

Over the course of one year (2023–2024), a total of 100 NS1 positive serum samples were collected. Analysis of these samples revealed that out of 100 samples, 65 samples tested positive for dengue virus through RT-PCR using consensus DENV primers D1 and D2.. Further, serotypes of the positive samples were also determined using type specific primers (TS1-F&R, TS2-F&R, TS3-F&R,TS4-F&R). Among the 65 positive samples, 63 tested positive for serotype 2 and two samples showed mix-serotype tested positive for both serotype 2 and 3, that indicated that serotype 2 was the predominant strain during 2023–24, (Fig. 1).

Out of these 100 samples, 65 were amplified successfully and observed using the gel documentation system following gel electrophoresis. As shown in **Supplementary Figure 1** (1 to 12), 63 samples showed bands at 403 bp, corresponding to serotype-2, Two samples displayed bands at 403 and 453 bp, corresponding to serotypes 2 and 3, indicating a mixed serotype as shown in **Supplementary Figure 2**. A 100 bp ladder was used as a reference.

### 3.2 Amplification of envelope gene

The amplification of the dengue virus envelope gene, approximately 1.4 kilobases (kb) in size, was successfully achieved after careful optimization of various PCR conditions, including denaturation, annealing, and extension temperatures, to ensure optimal amplification efficiency. Nested PCR was employed for this process, utilizing both specific inner and outer primers to enhance the specificity and yield of the desired product. The amplified PCR products were subjected to electrophoresis on a 1.5% agarose gel to separate and visualize the DNA fragments. A DNA ladder of 100 bp was used as a molecular weight marker to accurately determine the size of the amplified products. The gel was then visualized using a gel documentation system, enabling clear visualization of the DNA bands. Out of the 65 samples confirmed positive for DENV-2, only a limited number exhibited successful amplification of the envelope gene **Supplementary Figure 3**. This selective amplification highlights the effectiveness of the nested PCR approach under the optimized conditions.

### 3.3 *In-silico* characterization of envelope gene

#### 3.3.1 Multiple sequence alignment

**Fig. 2:**
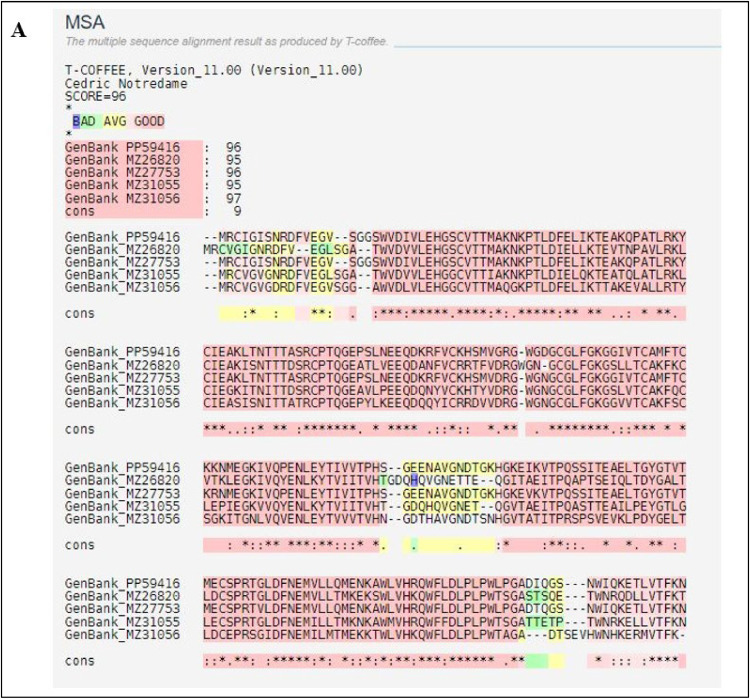

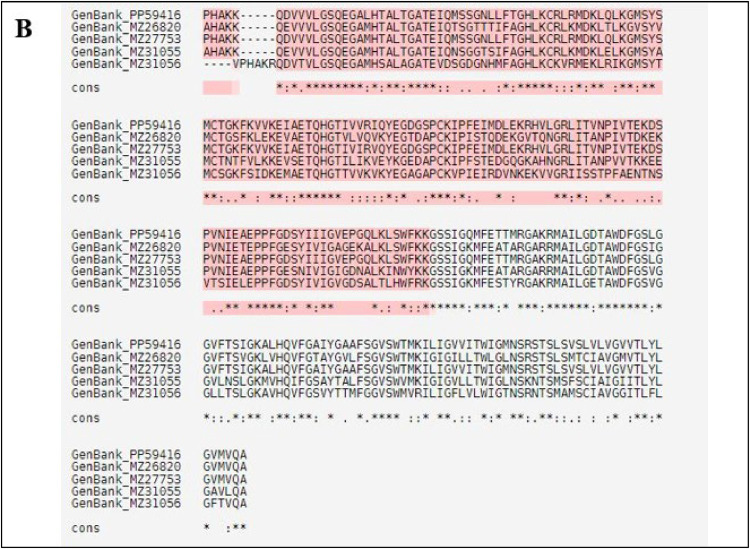
Multiple Sequence Alignment of Envelope Gene of Different Serotypes.

The MSA reveals extensive regions of high conservation, particularly in the N-terminal domain (positions 1–50), the central segment (positions 100–150), and the C-terminal domain (positions 200–250) were shown in Fig. 2. These highly conserved regions, depicted in red and marked by asterisks (*) in the consensus line, indicating the critical functional or structural roles. Partially conserved regions, denoted by colons (:) and periods (.), suggesting areas with potential functional flexibility.

The alignment yielded a score of 96, indicating a High quality of alignment. The alignment quality was categorized into three segments: 'High' (red) alignment score above 90, 'Moderate' (yellow) scoring between 70 and 89, and 'Low' (green) score below 70. A significant portion of the alignment classified as 'High,' reflecting considerable sequence conservation, while some segments were categorized as 'Moderate' and a few as 'Low.' The high-quality alignment, particularly in functionally significant motifs, underscores the evolutionary conservation of these regions. Variability was primarily observed in loop and linker regions, which typically experience less evolutionary pressure compared to core functional domains. This variability provides insights into the potential flexibility and adaptability of the protein sequences.

T-Coffee was chosen for multiple sequence alignment due to its ability to integrate both global and local alignment information, offering greater accuracy in aligning sequences with structural importance, such as the envelope protein sequences in our study. Its strength in aligning sequences with varying levels of conservation was essential for identifying conserved residues crucial for docking analysis.

Overall, the MSA of the gene isolated and related sequences from GenBank demonstrates substantial conservation, particularly in functionally significant motifs, suggesting evolutionary constraints. The observed variability suggests potential flexibility, while the high alignment quality confirms the dependability of these conserved regions. This analysis is integral to understanding the functional and structural aspects of these sequences, thereby facilitating further research into Dengue virus studies.

#### 3.3.2 Phylogenetic analysis of detected serotype

**Fig. 3:**
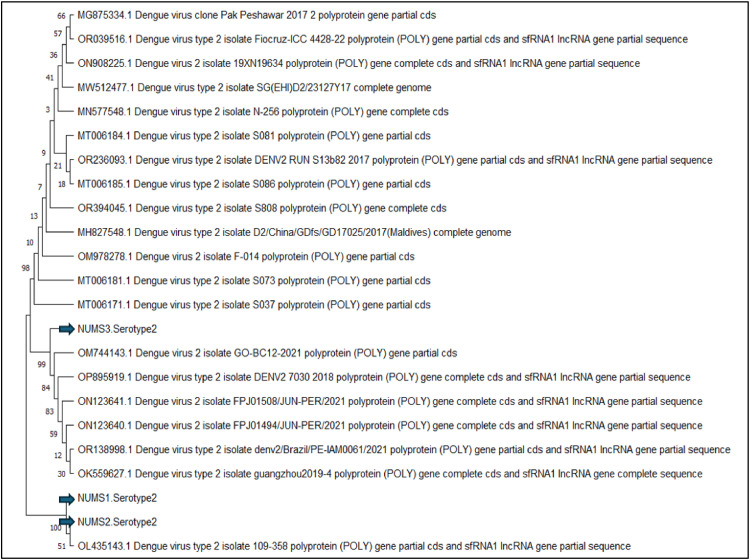
Phylogenetic tree using MEGA 11 (Neighbor-Joining, 100 bootstraps), with branches labeled by isolate accession numbers and countries, showing evolutionary relationships.

The phylogenetic tree illustrates the evolutionary relationships among various isolates of Dengue virus type 2 (DENV-2) Fig. 3. Each branch represents a distinct isolate, labeled with accession numbers and detailed descriptions indicating the isolate's origin, year of isolation, and the specific genetic regions sequenced, focusing on the partial coding sequence of the polyprotein region. The tree includes bootstrap values at each node, which provide a measure of confidence in the branching order; higher values signify stronger support for the associated branch's accuracy. Clustering patterns within the tree highlight genetic similarities, with closely grouped isolates being more genetically similar than those positioned further apart.

The isolates "NUMS3-Serotype2", "NUMS1-Serotype2″ and "NUMS2-Serotype2″ represent the gene sequences obtained in this study. These sequences were compared with existing ones to underscore their unique genetic features and their evolutionary relationship to other DENV-2 isolates. Understanding the genetic diversity, evolutionary dynamics, and geographic distribution of DENV-2 isolates depends on this analysis. DENV-2 sequences from various regions exhibit notable genetic similarities, highlighting the cross-border nature of the disease and the significance of regional cooperation in dengue control and prevention. This phylogenetic analysis provides valuable insights into the virus's evolutionary history and helps inform strategies for effective disease management.

#### 3.3.3 Homology modeling

In this study, homology modeling was used to construct the 3D structure of the Dengue virus envelope protein, providing crucial insights into its structural features. The protein sequence of the DENV-2 envelope gene (GenBank no: PP594161) was translated using the Expasy Translate tool, and then the 3D structure of the protein was modeled using the Swiss-Model server, which identified suitable templates from the Protein Data Bank (PDB) to generate a high-confidence structural model. The resulting models were visualized and analyzed using PyMOL, with distinct color-coding applied to different domains and sub-domains for clarity:

Domain 1, 2 and 3 are shown in yellow, red, and blue.
Stem/TM region are shown in green.

**Fig. 4:**
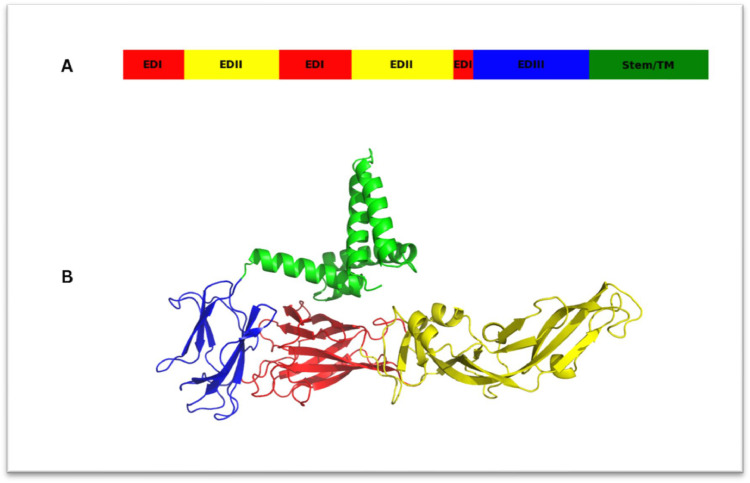
(A) Color bar represent different parts of the 3D Structure of DENV-2 (B) The three domains of the DENV-2 sE protein are shown: E-DI is colored red, E-DII is yellow, and E-DIII is blue. A 53-residue stem segment connects the stable sE fragment to the to the C-terminal transmembrane anchor.

This visualization allowed us to clearly differentiate the various functional regions of the protein (Fig. 4). This model structural insights are crucial for comprehending the interaction mechanisms and function of the protein.

The comprehensive structural model of our protein revealed critical regions that may interact with antiviral compounds. Specifically, the model highlighted potential binding sites for Arbidol and Quercetin, guiding future drug design efforts. These findings provide a foundation for further experimental validation and therapeutic development. By leveraging advanced bioinformatics tools, the structural architecture of the Dengue virus was elucidated that offering new perspectives on its role in viral infectivity and potential avenues for antiviral intervention.

#### 3.3.4 Molecular docking

##### 3.3.4.1 Docking with Arbidol

**Fig. 5:**
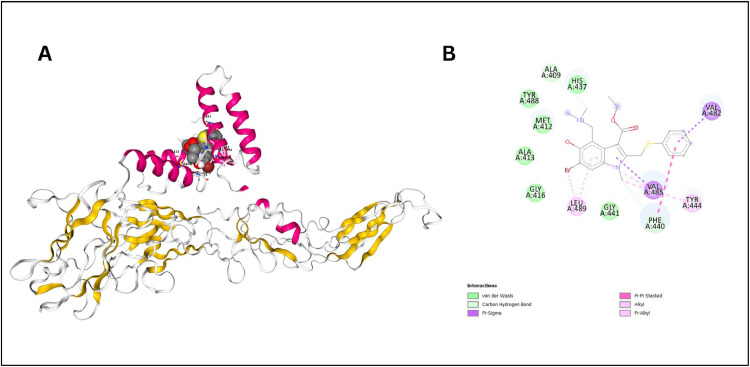
(A) 3D structure showing Arbidol nestled within a cavity of the dengue virus serotype-2 envelope protein, stabilized by surrounding α-helices and β-sheets. (B) 2D representation of residues around Arbidol, highlighting interactions within the binding pocket.

The docking study of Arbidol with the structural protein of the dengue virus, was conducted to evaluate the binding interactions and predict the pharmacokinetic properties of the drug. The results reveal that Arbidol forms a stable complex with the envelope protein, with a docking score of -7.2 kcal/mol, through various non-covalent interactions. Key interactions include van der Waals forces with residues such as ALA409, TYR488, and MET412, a carbon hydrogen bond with HIS437, Pi-Sigma interactions involving PHE440, and Pi-Pi stacked interactions with LEU489. Additionally, alkyl interactions with VAL482 and VAL485, and Pi-Alkyl interactions with PHE440 and TYR444, further stabilize the binding. The 2D structural visualization confirmed Arbidol location within the binding pocket, emphasizing the role of these interactions in the binding affinity (Fig. 5B).

The 3D image shows Arbidol nestled within a specific cavity of the protein **(**Fig. 5A). Surrounding α-helices and β-sheets were key elements that stabilized the molecule through multiple interactions. Arbidol predominantly interacted with (residues 396–495) of the envelope protein, including VAL482, PHE440, and LEU489, which were crucial for maintaining the protein's structure and function. The binding pocket features both hydrophobic and hydrophilic residues, with various π interactions (Pi-Sigma, Pi-Pi stacked, Pi-Alkyl) highlighted the importance of aromatic residues in stabilizing the compound within the pocket. These interactions suggested that Arbidol effectively binds to the virus envelope protein, potentially inhibiting its function, which supports its potential as an antiviral agent against dengue virus.

###### 3.3.4.1.1 ADMET Properties of Arbidol

Arbidol exhibited good intestinal absorption (88.296%) and moderate Caco-2 permeability (0.834), but low skin permeability (-2.732). In terms of distribution, it has a volume of distribution (VDss) of 0.726 L/kg, limited permeability of the blood-brain barrier (0.031) and the central nervous system (-2.191). Metabolically, Arbidol is a substrate for CYP3A4 but not for CYP2D6, and it inhibits several cytochrome P450 enzymes, indicating potential drug interactions. It has a total clearance rate of 0.688 log ml/min/kg and no interaction with renal OCT2. Regarding toxicity, Arbidol does not showed AMES toxicity but has a moderate maximum tolerated dose (0.333 log mg/kg/day) in humans, with potential hepatotoxicity and hERG II inhibition risks. Its acute oral toxicity (LD50) in rats is 2.958 mol/kg, suggesting a relatively safe profile at standard doses. These findings emphasize Arbidol's potential as a therapeutic agent while noting important considerations for its metabolic interactions and toxicity risks, laying the groundwork for further experimental validation and antiviral drug development. The predicted ADMET properties of Arbidol, highlighting several key findings are summarized (Table 3).

**Table 3:** Provides the details of Arbidol predicted ADMET.

Property	Predicted Value	Unit	Model Name
**Absorption**			
Absorption	0.83	Numeric (log Papp in 10^-6 cm/s)	Ca:02 permeability
Absorption	-3.98	Numeric log mol/L)	Water solubility
Absorption	Yes	Categorical	P-glycoprotein substrate
Absorption	88.29	Numeric (% Absorbed)	Intestinal absorption (human)
Absorption	2.73	Numeric (log Kp)	Skin Permeability
Absorption	Yes	Categorical	P-glycoprotein II inhibitor
Absorption	Yes	Categorical	2-glycoprotein I inhibitor
**Distribution**			
Distribution	0.72	Numeric log L/kg)	VDss (human)
Distribution	0.03	Numeric (log BB)	Bas permeability
Distribution	0.12	Numeric (Fu)	Fraction unbound (human)
Distribution	-2.0	Numeric (log PS)	CNS permeability
**Metabolism**			
Metabolism	No	Categorical	CYP2D6 substrate
Metabolism	Yes	Categorical	CYP3A4 substrate
Metabolism	Yes	Categorical	CYP2D6 inhibition
Metabolism	No	Categorical	CYP1A2 inhibitor
Metabolism	No	Categorical	CYP2C9 inhibitor
Metabolism	Yes	Categorical	CYP2C19 inhibition
Metabolism	Yes	Categorical	CfP3A4 inhibitor
**Excretion**			
Excretion	0.68	Numeric (log ml/min/kg)	Total Clearance
Excretion	No	Categorical	Renal Clearance
**Toxicity**			
Toxicity	Yes	Categorical	Hepatotoxicity
Toxicity	0.33	Numeric (log mg/kg/day)	Max. tolerated dose (human)
Toxicity	No	Categorical	AMES toxicity
Toxicity	No	Categorical	hERG I inhibitor
Toxicity	Yes	Categorical	hERG II inhibitor
Toxicity	2.95	Numeric (mol/kg)	Oral Rat Acute Toxicity (LD50)
Toxicity	0.73	Numeric (log mg/kg_bw/day)	Oral Rat Chronic Toxicity (LOAEL)
Toxicity	0.29	Numeric (log ug/L)	T.Pyriformis toxicity
Toxicity	No	Categorical	Skin Sensitization
Toxicity	-0.12	Numeric (Log mM)	Minnow toxicity

##### 3.3.4.2 Docking With Quercetin

**Fig. 6:**
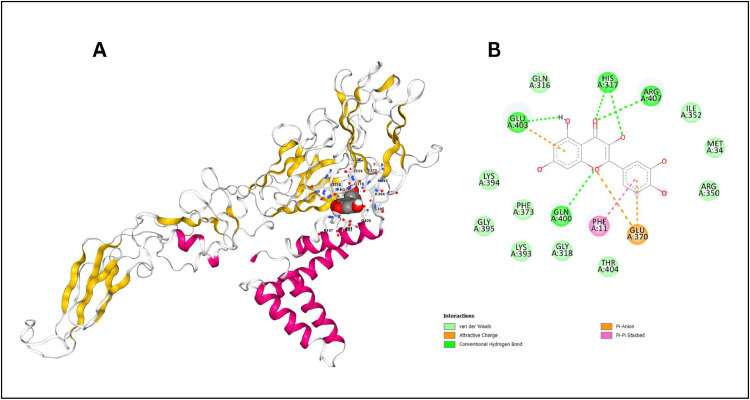
(A) 3D structure showing Quercetin nestled within a cavity of the dengue virus serotype-2 envelope protein, stabilized by surrounding α-helices and β-sheets. (B) 2D representation of residues around Quercetin, highlighting interactions within the binding pocket.

To elucidate the binding dynamics between quercetin and the dengue virus envelope protein, computational docking analysis were conducted. The simulations demonstrated a strong interaction profile, with a docking score of -8.2 kcal/mol, showcasing significant binding affinity between the ligand and the protein. The binding poses and dynamics are illustrated in Fig. 6A.

Quercetin binds within a specific pocket of the virus envelope protein, engaging various types of interactions, including hydrogen bonds, pi-anion interactions, pi-pi stacking interactions, and van der Waals forces. The visual representation in Fig. 6B highlights the ligand position and interactions with key protein residues.

Key residues involved in binding include HIS 317 and GLN 316, which form hydrogen bonds with quercetin, stabilizing its position within the binding pocket. GLU 370 participates in pi-anion interactions with the aromatic rings of quercetin, while PHE 11 was crucial for pi-pi stacking interactions, essential for stabilizing the aromatic system of the ligand. Additionally, residues such as GLN 400, GLU 403, HIS 317, and GLN 316 were observed to have involvement in van der Waals interactions, helping to maintain the ligand within the binding site. Other residues like LYS 394, GLY 395, and ARG 407 were observed in proximity and may contribute to the overall binding through weaker interactions. These interactions are critical for the stable binding of quercetin and may influence its potential inhibitory effects on the protein function.

###### 3.3.4.2.1 ADMET properties of quercetin

The ADMET profile of quercetin was analyzed to assess its pharmacokinetic and safety characteristics (Table 4). Quercetin has low skin permeability but high intestinal absorption (77.20%) due to its moderate Caco-2 permeability and water solubility. It is a substrate for P-glycoprotein but does not inhibit P-glycoprotein I or II. The volume of distribution is extensive (1.55 log L/kg), and quercetin shows moderate plasma binding (fraction unbound of 0.20) with limited blood-brain barrier permeability. It does not serve as a substrate for CYP2D6 or CYP3A4, inhibits CYP1A2, and has no impact on other CYP enzymes. Its total clearance is relatively low (0.40 log ml/min/kg), and it does not interact with the renal OCT2 substrate. Quercetin shows no AMES toxicity, has a high tolerated dose in humans (0.49 log mg/kg/day), and does not inhibit hERG channels. Its oral rat acute toxicity (LD50) is 2.47 mol/kg, indicating a safe profile at typical doses. Additionally, quercetin does not exhibit hepatotoxicity, skin sensitization, or significant toxicity to Tetrahymena pyriformis or minnows. This profile highlights quercetin's potential as a compound that is safe and acceptable, making it a suitable candidate for additional pharmaceutical research.

**Table 4:** Provides the details of Quercetin predicted ADMET properties.

Property	Predicted Value	Unit	Model
Absorption			
Absorption	77.20	% Absorbed	Intestinal absorption (human)
Absorption	-2.92	log mol/L	Water solubility
Absorption	-0.22	Papp in 10^-6 cm/s	Caco-2 permeability
Absorption	No	Categorical	P-glycoprotein I inhibitor
Absorption	-2.73	log Kp	Skin permeability
Absorption	Yes	Categorical	P-glycoprotein substrate
Absorption	No	Categorical	P-glycoprotein II inhibitor
**Distribution**			
Distribution	1.55	log L/kg	VDss (human)
Distribution	-3.06	log PS	CNS permeability
Distribution	0.20	Fu	Fraction unbound (human)
Distribution	-1.09	log BB	BBB permeability
**Metabolism**			
Metabolism	No	Categorical	CYP1A2 inhibitor
Metabolism	Yes	Categorical	CYP3A4 inhibitor
Metabolism	No	Categorical	CYP2C19 inhibitor
Metabolism	No	Categorical	CYP2C9 inhibitor
Metabolism	No	Categorical	CYP2D6 inhibitor
Metabolism	No	Categorical	CYP2D6 substrate
Metabolism	No	Categorical	CYP3A4 substrate
**Excretion**			
Excretion	0.40	log ml/min/kg	Total Clearance
Excretion	No	Categorical	Renal OCT2 substrate
**Toxicity**			
Toxicity	No	Categorical	Hepatotoxicity
Toxicity	No	Categorical	AMES toxicity
Toxicity	0.49	log mg/kg/day	Max. tolerated dose (human)
Toxicity	No	Categorical	hERG I inhibitor
Toxicity	No	Categorical	hERG II inhibitor
Toxicity	2.47	mol/kg	Oral Rat Acute Toxicity (LD50)
Toxicity	2.61	kg_bw/day	Oral Rat Chronic Toxicity (LOAEL)
Toxicity	3.72	mM	Minnow toxicity
Toxicity	No	Categorical	Skin Sensitization
Toxicity	0.28	ug/L	T. Pyriformis toxicity

#### 3.3.5 Comparison of BepiPred-3.0 scores across all serotypes

To compare the BepiPred-3.0 scores across all serotypes, the average scores for each amino acid residue across all available serotypes were gathered. This data allows us to analyze and compare the scores to identify any patterns or differences. By analyzing this data, the common patterns or differences in antigenicity or immunogenicity among different serotypes can be identified.

The ANOVA test was performed to compare the BepiPred 3.0 scores among the four dengue virus serotypes. This test yielded both an F-value and a p-value, where the F-value represents the ratio of variance between group means to the variance within the groups, and the p-value indicates whether the observed differences are statistically significant. The purpose of the analysis was to determine the significance of the variations in epitope predictions amongst serotypes.

**Fig. 7:**
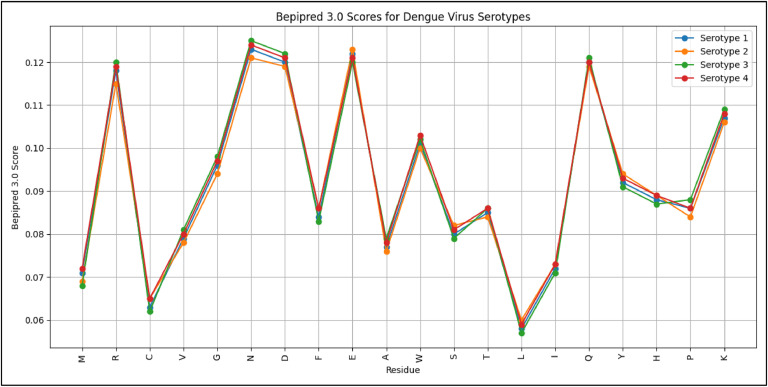
Line plot illustrating the Bepipred 3.0 scores for each residue across the four dengue virus serotypes.

To visualize these results, a line plot was generated that Fig. 7, illustrated the values for each residue across the four serotypes. The interpretation of this data revealed that the scores for each residue are relatively consistent across serotypes, suggesting similar predicted epitope regions. High-scoring residues such as R, N, E, and Q consistently show elevated scores, indicating their potential importance as epitopes. Conversely, residues like C, L, and A exhibit lower scores across all serotypes, implying they are less likely to be significant epitopes.

The mean BepiPred 3.0 scores for the residues were very similar across the four dengue virus serotypes, with an average value of approximately 0.091 and comparable standard deviations, indicating consistent variability in the scores. The ANOVA test resulted in an F-value of 0.003262 and a p-value of 0.998696. Since the p-value is significantly higher than 0.05, that suggested that there are no statistically significant differences in the BepiPred 3.0 scores among the serotypes. This analysis provides insights into the immunogenic potential of amino acid residues across different serotypes, indicating that identified potential epitopes might be universally relevant. Understanding these patterns can aid in designing vaccines and therapeutic antibodies by targeting the most immunogenic residues and considering conserved and variable regions. Further experimental validation is needed to confirm these computational predictions and explore their implications for vaccine development and immunotherapy.

## 4 Discussion

Dengue fever, an arthropod-borne viral illness from the *Flaviviridae* family, poses a significant global health threat, affecting over 128 countries and putting approximately 3.97 billion people at risk yearly (Dash et al., 2013). Tropical and subtropical regions are particularly affected by this disease, with Asia bearing the greatest burden. Approximately 75% of cases worldwide are found in countries like Thailand, Indonesia, and the Philippines, whereas South Asia, which includes Bangladesh and Pakistan, has a lower seroprevalence of dengue than does Southeast Asia. (Ali et al., 2022). In Pakistan, dengue has been a persistent problem since 1994, with severe outbreaks becoming more frequent since 2006, leading to notable rates of morbidity and mortality (Chan et al., 1995; Jamil et al., 2007). The economic impact is also significant, with Southeast Asian countries alone incurring costs exceeding $1 billion annually due to dengue (Shepard et al., 2013). Distinct 4 serotypes of the dengue virus included are DENV-1 to 4, which causes dengue fever (Ali et al., 2022).

In the current study 100 NS1-positive serum samples were analyzed from 2023 and 2024, that resulted in the detection of DENV-2 in 63 samples and DENV-2 and DENV-3 in two samples, indicating mixed infections. This aligns with previous research, which identified DENV-2 as the predominant serotype in Pakistan (Fatima et al., 2011) and confirmed its prevalence in recent outbreaks (Umair et al., 2023). The historical data from Karachi and other regions further supports the ongoing dominance of DENV-2 (Cobelens et al., 2002; Khanani et al., 2011; Qureshi et al., 1997). The robust methodology employed, including nested PCR with type-specific primers, validated the predominant presence of DENV-2 and provided valuable insights into dengue virus epidemiology.

This study focused extensively on the envelope (E) gene of the dengue virus, a critical component for viral entry into host cells and a key target for immune responses. The envelope protein is essential for the virus's attachment to host cell receptors and the subsequent fusion of viral and cellular membranes, which facilitates viral entry. Given its central role in the dengue virus life cycle, the envelope protein has been a primary target for various molecular and therapeutic studies.

In current research, a rigorous nested PCR approach was employed to amplify and characterize the envelope gene across multiple samples. Despite the large number of samples analyzed, only a subset yielded positive results for the envelope gene. This outcome underscores the genetic variability and the potential challenges in consistently amplifying and detecting viral components. Important information about the genetic makeup and genetic variability of the dengue virus in the area was gained from the successful amplification of the envelope gene in these samples.

Previous studies have extensively characterized the envelope gene, revealing its role in the virus's antigenic properties and its interaction with the host immune system. For instance, Li et al. (2020) utilized real-time PCR assays to explore the dynamics of the envelope gene during the 2018 outbreak in Hunan Province, China. Similarly, Jiang et al. (2018) conducted a comprehensive analysis of the dengue virus genome, including the envelope gene, to understand its mutations and recombinations. (Bai et al., 2013) focused on the envelope gene's role in vaccine development and its impact on virus-host interactions. These studies contribute to a broader understanding of the envelope gene's molecular characteristics and its significance in dengue virus pathogenesis.

Our findings highlighted the predominance of DENV-2, that are consistent with historical and recent research (Ali et al., 2022). The envelope gene genetic diversity and evolutionary dynamics as observed in our study emphasize the need for ongoing molecular characterization and surveillance. This continuous monitoring is vital for understanding shifts in serotype prevalence and the emergence of new genotypes, which can significantly impact vaccine development and therapeutic strategies. By elucidating the envelope gene's role and variability, our study contributes valuable data to inform public health measures and enhance our capacity to combat dengue fever effectively.

Our computational docking analysis revealed that quercetin binds with high affinity to key residues in the dengue virus envelope protein, notably HIS 317, GLN 316, GLU 370, and PHE 11. These residues play a pivotal role in stabilizing quercetin within the binding pocket through hydrogen bonds and pi-pi stacking interactions. Specifically, HIS 317 and GLN 316 form strong hydrogen bonds, while GLU 370 engages in pi-anion interactions with quercetin's aromatic rings, and PHE 11 contributes to pi-pi stacking, collectively ensuring the ligand's stable positioning within the protein. These interactions emphasize the functional importance of these conserved residues across DENV-2 and potentially other dengue serotypes.

Our findings align with prior studies on quercetin derivatives, such as quercetin-7-O-rutinoside, which also forms hydrogen bonds with NS2B/NS3 protease residues like Asp71, Lys74, and Trp83. This highlights quercetin's broad ability to target critical viral proteins. The hydrogen bonding and pi-pi stacking observed in the envelope protein are analogous to the hydrophobic and hydrogen bond interactions identified in the protease, suggesting that quercetin's antiviral potential may extend beyond the envelope protein to other viral components essential for replication (Shimu et al., 2022).

While computational docking provides valuable insights into the potential binding interactions of quercetin with the DENV-2 envelope protein, it has inherent limitations. Docking studies are predictive and do not account for the full complexity of biological environments, such as protein flexibility, cellular conditions, and pharmacokinetics. Therefore, experimental validation, such as in vitro or in vivo studies, would be necessary to confirm quercetin's antiviral effects and its efficacy against DENV-2.

## 5 Conclusion

This study successfully identified the predominant dengue virus serotype (DENV-2) circulating in Pakistan during 2023–2024. The comprehensive characterization of the envelope gene provided valuable insights into the virus molecular structure and function. The high sequence similarity to DENV-2 strains from regions such as India, China, and Singapore underscores the widespread distribution and genetic stability of this serotype. These results are critical for developing public health policies and strengthening our ability to fight dengue virus infections locally and worldwide. In addition to molecular characterization, computational docking studies with antiviral compounds like Arbidol and Quercetin indicated potential therapeutic interactions. These results suggest promising leads for antiviral therapy development. The identification of potential B-cell epitopes through BepiPred-3.0 analysis is a significant step toward future vaccine development.

## Servers and databases

The below servers and databases used

1. Nucleotide BLAST (https://blast.ncbi.nlm.nih.gov/Blast.cgi)
2. Expasy Translate Tool (https://web.expasy.org/translate/)
3. T-Coffee Server (https://tcoffee.crg.eu/)
4. Swiss Model Server (https://swissmodel.expasy.org/)
5. Pub Chem (https://pubchem.ncbi.nlm.nih.gov/)
6. CB-Dock (http://clab.labshare.cn/cb-dock/)
7. BepPred 3.0 (https://services.healthtech.dtu.dk/services/BepiPred-3.0/)

## Ethical approval

This study protocol was approved by the Ethics Review Committee (ERC) and by Institutional Review Board (IRB) of the National University of Medical Sciences (NUMS), Rawalpindi, Punjab, Pakistan.

## CRediT authorship contribution statement

**Haidar Ali:** Writing – review & editing, Writing – original draft, Software, Resources, Methodology, Investigation, Formal analysis, Data curation. **Iffat Saleem:** Writing – review & editing, Methodology, Formal analysis, Data curation. **Muhammad Saad Ahmed:** Writing – review & editing, Writing – original draft, Data curation. **Deeba Amraiz:** Writing – review & editing, Resources, Formal analysis. **Imran Shahid:** Writing – review & editing, Formal analysis. **Eman A. Al-Shahari:** Data curation, Funding acquisition, Resources. **Jing Yang:** Data curation, Resources, Writing – review & editing. **Liaqat Ali:** Writing – review & editing, Writing – original draft, Supervision, Resources, Project administration, Investigation, Funding acquisition, Formal analysis, Data curation, Conceptualization.

## Declaration of competing interest

The authors declare that they have no known competing financial interests or personal relationships that could have appeared to influence the work reported in this paper.

## Data Availability

Data will be made available on request.

## Appendix Supplementary materials

Image, application 1

## References

[bib0001] Ali L., Gul Z., Ijaz A., Khalid N., Zeb F., Afzal S., Ullah A., Subhan F., Ahmed S. (2022). An overview of dengue viral infection circulating in Pakistan. J. Vector. Borne Dis..

[bib0002] Bai Z., Liu L.C., Jiang L.Y., Liu Q., Cao Y.M., Xu Y., Jing Q.L., Luo L., Yang Z.C., Jiang Y.Q., Chen W., Di B. (2013). Complete genome sequence of dengue virus serotype 3 from Guangzhou, China. Genome Announc..

[bib0003] Bhatnagar P., Sreekanth G.P., Murali-Krishna K., Chandele A., Sitaraman R. (2021). Dengue virus non-structural protein 5 as a versatile, multi-functional effector in host–pathogen interactions. Front. Cell Infect. Microbiol..

[bib0004] Bhatt S., Gething P.W., Brady O.J., Messina J.P., Farlow A.W., Moyes C.L., Drake J.M., Brownstein J.S., Hoen A.G., Sankoh O., Myers M.F., George D.B., Jaenisch T., William Wint G.R., Simmons C.P., Scott T.W., Farrar J.J., Hay S.I. (2013). The global distribution and burden of dengue. Nature.

[bib0005] Bressanelli S., Stiasny K., Allison S.L., Stura E.A., Duquerroy S., Lescar J., Heinz F.X., Rey F.A. (2004). Structure of a flavivirus envelope glycoprotein in its low-pH-induced membrane fusion conformation. EMBO J..

[bib0006] Chambers T.J., Hahn C.S., Galler R., Rice C.M. (1990). Flavivirus genome organization, expression, and replication. Annu. Rev. Microbiol..

[bib0007] Chan Y.C., Salahuddin N.I., Khan J., Tan H.C., Seah C.L.K., Li J., Chow V.T.K. (1995). Dengue haemorrhagic fever outbreak in Karachi, Pakistan, 1994. Trans. R. Soc. Trop. Med. Hyg..

[bib0008] Cobelens F.G.J., Groen J., Osterhaus A.D.M.E., Leentvaar-Kuipers A., Wertheim-Van Dillen P.M.E., Kager P.A. (2002). Incidence and risk factors of probable dengue virus infection among Dutch travellers to Asia. Trop. Med. Int. Health.

[bib39] Dash P.K., Sharma S., Soni M., Agarwal A., Parida M., Rao P.V.L. (2013). Complete genome sequencing and evolutionary analysis of Indian isolates of Dengue virus type 2. Biochem. Biophys. Res. Commun..

[bib0009] de Wispelaere M., Lian W., Potisopon S., Li P.C., Jang J., Ficarro S.B., Clark M.J., Zhu X., Kaplan J.B., Pitts J.D., Wales T.E., Wang J., Engen J.R., Marto J.A., Gray N.S., Yang P.L. (2018). Inhibition of flaviviruses by targeting a conserved pocket on the viral envelope protein. Cell Chem. Biol..

[bib0010] Dethoff E.A., Boerneke M.A., Gokhale N.S., Muhire B.M., Martin D.P., Sacco M.T., McFadden M.J., Weinstein J.B., Messer W.B., Horner S.M., Weeks K.M. (2018). Pervasive tertiary structure in the dengue virus RNA genome. Proc. Natl. Acad. Sci. USA.

[bib0011] Fatima Z., Idrees M., Bajwa M.A., Tahir Z., Ullah O., Zia M.Q., Hussain A., Akram M., Khubaib B., Afzal S., Munir S., Saleem S., Rauff B., Badar S., Naudhani M., Butt S., Aftab M., Ali L., Ali M. (2011). Serotype and genotype analysis of dengue virus by sequencing followed by phylogenetic analysis using samples from three mini outbreaks-2007-2009 in Pakistan. BMC Microbiol..

[bib0012] Guo C., Zhou Z., Wen Z., Liu Y., Zeng C., Xiao D., Ou M., Han Y., Huang S., Liu D., Ye X., Zou X., Wu J., Wang H., Zeng E.Y., Jing C., Yang G. (2017). Global epidemiology of dengue outbreaks in 1990–2015: a systematic review and meta-analysis. Front. Cell Infect. Microbiol..

[bib0013] Harapan H., Michie A., Sasmono R.T., Imrie A. (2020). Dengue: a mini review. Viruses.

[bib0014] Jamil B., Hasan R., Zafar A., Bewley K., Chamberlain J., Mioulet V., Rowlands M., Hewson R. (2007). Dengue virus serotype 3, Karachi, Pakistan [9]. Emerg. Infect Dis..

[bib0015] Jiang L., Ma D., Ye C., Li L., Li X., Yang J., Zhao Y., Xi J., Wang X., Chen J., Pan Y., Shan X., Sun Q. (2018). Molecular characterization of dengue virus serotype 2 cosmospolitan genotype from 2015 dengue outbreak in Yunnan, China. Front. Cell Infect. Microbiol..

[bib0016] Khan E., Hasan R., Mehraj V., Nasir A., Siddiqui J., Hewson R. (2008). Co-circulations of two genotypes of dengue virus in 2006 out-break of dengue hemorrhagic fever in Karachi, Pakistan. J. Clinic. Virol..

[bib0017] Khanani M.R., Arif A., Shaikh R. (2011). Dengue in Pakistan : journey from a disease free to a Hyper Endemic Nation. J. Dow Univ. Health Sci. Karachi.

[bib0018] Klein D.E., Choi J.L., Harrison S.C. (2013). Structure of a dengue virus envelope protein late-stage fusion intermediate. J. Virol..

[bib37] Lanciotti R.S., Calisher C.H., Gubler D.J., Chang G.J., Vorndam A.V. (1992). Rapid detection and typing of dengue viruses from clinical samples by using reverse transcriptase-polymerase chain reaction. J. Clin. Microbiol..

[bib0019] Li X., Guan B., Su T., Liu W., Chen M., Bin Waleed K., Guan X., Gary T., Zhu Z. (2020). Impact of cardiovascular disease and cardiac injury on in-hospital mortality in patients with COVID-19: a systematic review and meta-analysis. Heart.

[bib0020] Limjindaporn T., Wongwiwat W., Noisakran S., Srisawat C., Netsawang J., Puttikhunt C., Kasinrerk W., Avirutnan P., Thiemmeca S., Sriburi R., Sittisombut N., Malasit P., Yenchitsomanus P.thai (2009). Interaction of dengue virus envelope protein with endoplasmic reticulum-resident chaperones facilitates dengue virus production. Biochem. Biophys. Res. Commun..

[bib0021] Modis Y., Ogata S., Clements D., Harrison S.C. (2005). Variable surface epitopes in the crystal structure of dengue virus type 3 envelope glycoprotein. J. Virol..

[bib0022] Mukhopadhyay S., Kuhn R.J., Rossmann M.G. (2005). A structural perspective of the Flavivirus life cycle. Nat. Rev. Microbiol..

[bib0023] Murugesan, A., Manoharan, M., 2020. Chapter 16 - Dengue Virus, in: Emerging and Reemerging Viral Pathogens.

[bib0024] Ng R.J., Chong Z.L., Mutalip M.H.A., Ng C.W. (2022). Dengue seroprevalence and factors associated with dengue seropositivity in Petaling District, Malaysia. Int. J. Environ. Res. Public Health.

[bib0025] Patil J.A., Alagarasu K., Kakade M.B., More A.M., Gadekar K.A., Jadhav S.M., Parashar D., Shah P.S. (2018). Emergence of dengue virus type 1 and type 3 as dominant serotypes during 2017 in Pune and Nashik regions of Maharashtra, Western India. Infect. Genet. Evol..

[bib0026] Qureshi J.A., Notta N.J., Salahuddin N., Zaman V., Khan J.A. (1997). An epidemic of dengue fever in Karachi - Associated clinical manifestations. J. Pak. Med. Assoc..

[bib0027] Roehrig J.T., Hunt A.R., Johnson A.J., Hawkes R.A. (1989). Synthetic peptides derived from the deduced amino acid sequence of the E-glycoprotein of Murray Valley encephalitis virus elicit antiviral antibody. Virology.

[bib0028] Scaturro P., Cortese M., Chatel-Chaix L., Fischl W., Bartenschlager R. (2015). Dengue virus non-structural protein 1 modulates infectious particle production via interaction with the structural proteins. PLoS. Pathog..

[bib38] Shepard D.S., Undurraga E.A., Halasa Y.A. (2013). Economic and Disease Burden of Dengue in Southeast Asia. PLoS Negl. Trop. Dis..

[bib0029] Shimu M.S.S., Mahmud S., Tallei T.E., Sami S.A., Adam A.A., Acharjee U.K., Paul G.K., Emran T.Bin, Zaman S., Uddin M.S., Saleh M.A., Alshehri S., Ghoneim M.M., Alruwali M., Obaidullah A.J., Jui N.R., Kim J., Kim B. (2022). Phytochemical compound screening to identify novel small molecules against dengue virus: a docking and dynamics study. Molecules.

[bib0030] Umair M., Haider S.A., Rehman Z., Jamal Z., Ali Q., Hakim R., Bibi S., Ikram A., Salman M. (2023). Genomic characterization of dengue virus outbreak in 2022 from Pakistan. Vaccines.

[bib0031] Usman M. (2018). Evaluation of bio-efficacy and residual activity of pyriproxyfen against field collected aedes aegypti and aedes albopictus from Gujranwala (Punjab), Pakistan. Int. J. Infect. Dis..

[bib0032] Yousaf M.Z., Siddique A., Ashfaq U.A., Ali M. (2018). Scenario of dengue infection & its control in Pakistan: an up-date and way forward. Asian Pac. J. Trop. Med..

[bib0033] Zhang Q., Hunke C., Yau Y.H., Seow V., Lee S., Tanner L.B., Guan X.L., Wenk M.R., Fibriansah G., Chew P.L., Kukkaro P., Biuković G., Shi P.Y., Shochat S.G., Grüber G., Lok S.M. (2012). The stem region of premembrane protein plays an important role in the virus surface protein rearrangement during dengue maturation. J. Biol. Chem..

[bib0034] Zhang W., Chipman P.R., Corver J., Johnson P.R., Zhang Y., Mukhopadhyay S., Baker T.S., Strauss J.H., Rossmann M.G., Kuhn R.J. (2003). Visualization of membrane protein domains by cryo-electron microscopy of dengue virus. Nat. Struct. Biol..

[bib0035] Zhang X., Sheng J., Plevka P., Kuhn R.J., Diamond M.S., Rossmann M.G. (2013). Dengue structure differs at the temperatures of its human and mosquito hosts. Proc. Natl. Acad. Sci. USA.

[bib0036] Zhang Y., Zhang W., Ogata S., Clements D., Strauss J.H., Baker T.S., Kuhn R.J., Rossmann M.G. (2004). Conformational changes of the flavivirus E glycoprotein. Structure.

