# Supplementary material for: Dominance of dengue virus serotype-2 in Pakistan (2023–2024): Molecular characterization of the envelope gene and exploration of antiviral targets
Source: Virus Res. 2024 Nov 23;350:199497. doi: 10.1016/j.virusres.2024.199497 (PMC11625376; doi:10.1016/j.virusres.2024.199497)
Supplement: Supplementary file 1 [file mmc1.docx]

**Supplementary Materials**

**Supplementary Figure 1**


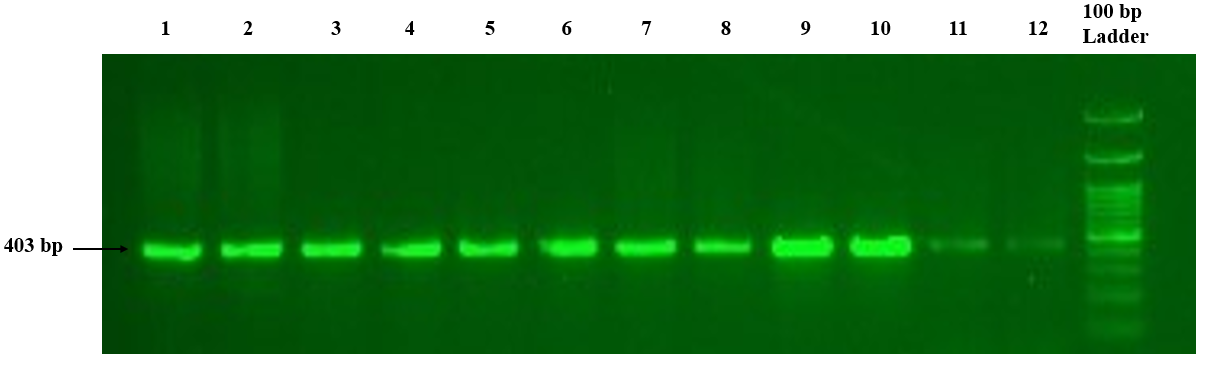


**Supplementary Figure 1:** Gel picture of PCR amplification of a Dengue Virus Serotype 2.

**Supplementary Figure 2**


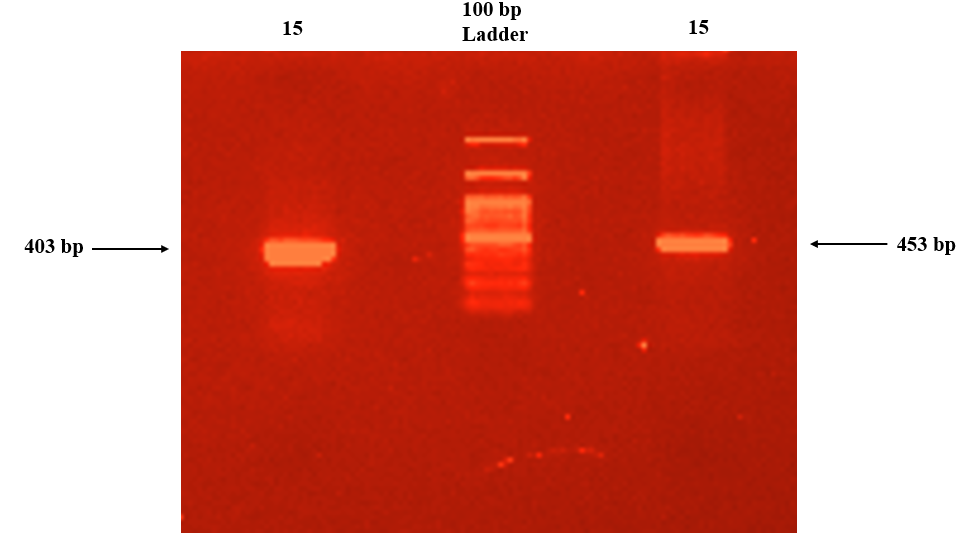


**Supplementary Figure 2**: Gel picture showing Mix-serotype in PCR amplification of Dengue Serotype 2 and 3.

**Supplementary Figure 3**


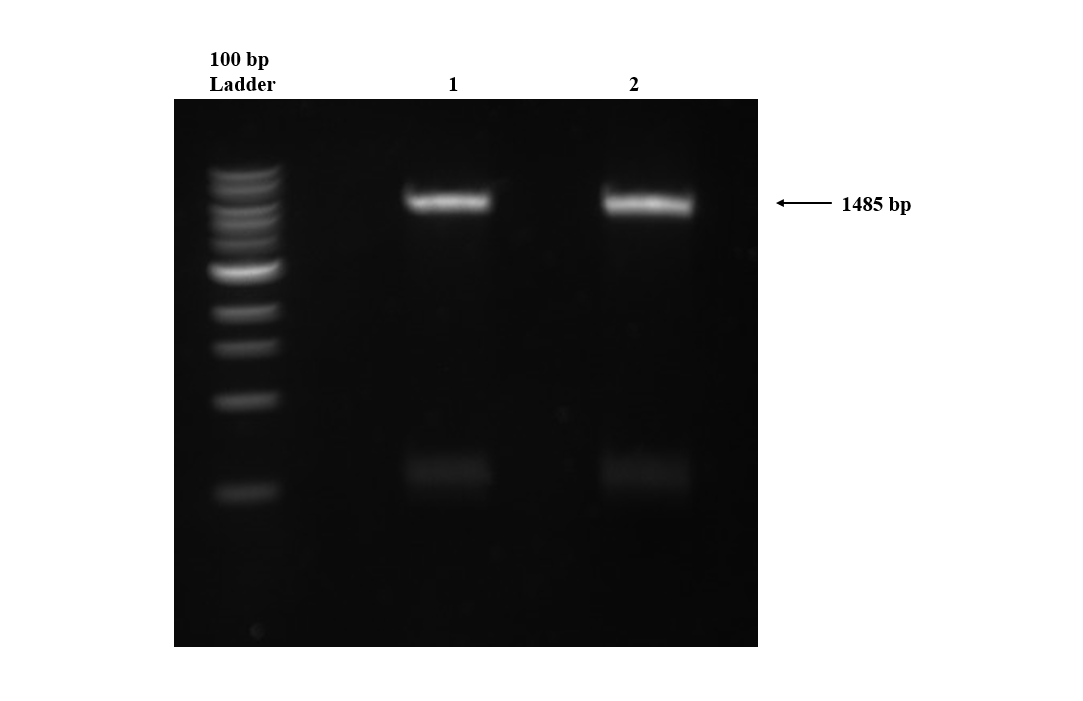


**Supplementary Figure 3**: Pictorial representation of amplified Envelope gene and size of the gene is 1485 bp with 100 bp Ladder as a reference.
